# N^6^-methyladenosine (m^6^A) reader IGF2BP1 facilitates clear-cell renal cell carcinoma aerobic glycolysis

**DOI:** 10.7717/peerj.14591

**Published:** 2023-01-18

**Authors:** Bao Yuan, Jin Zhou

**Affiliations:** 1 Tianjin Beichen Traditional Chinese Medicine Hospital, Tianjin, China; 2 NHC Key Laboratory of Hormones and Development, Chu Hsien-I Memorial Hospital and Tianjin Institute of Endocrinology, Tianjin Key Laboratory of Metabolic Diseases, Tianjin Medical University, Tianjin, China

**Keywords:** ccRCC, m6A, Aerobic glycolysis

## Abstract

Emerging articles have reported that N^6^-methyladenosine (m^6^A) modification is mainly involved in clear-cell renal cell carcinoma (ccRCC) tumorigenesis. However, the regulatory mechanisms of m^6^A reader IGF2BP1 involved in ccRCC tumor energy metabolism are currently unknown. Results showed that the m^6^A reader IGF2BP1 exhibited significantly higher expression in ccRCC cells. Functionally, results by gain/loss functional assays indicated that IGF2BP1 promoted the glycolytic characteristics, including glucose uptake, lactate production and extracellular acidification rate (ECAR). Mechanistically, IGF2BP1 recognized the m^6^A modified sites on LDHA mRNA and enhanced its mRNA stability, thereby accelerating tumor energy metabolism. Thus, our work reveals a novel facet of the m^6^A that promoted mRNA stability and highlighted the functional importance of IGF2BP1 as m^6^A readers in post-transcriptional gene regulation.

## Introduction

Renal cell carcinoma (RCC) acts as the second leading cause of urological malignant neoplasm-related death and tens of thousands of newly diagnosed cases are reported by global statistics (Cao et al., 2022; Kench et al., 2022). More precisely, clear cell renal cell carcinoma (ccRCC) is a higher invasive subtype of RCC, comprising 80–90% of RCC patients (Liu et al., 2022b; Pei et al., 2022). Unfortunately, the chemotherapy or radiotherapy is largely ineffective for all renal cancer subtypes (Vassiliou et al., 2022; Yin & Zheng, 2022). Therefore, the immunotherapy or targeted therapy is a very valuable alternative strategy.

N^6^-methyladenosine (m^6^A) modification is firstly described in 1971 years, which has shown critical roles in mRNA metabolism and become focus recently (Chen et al., 2022; Li et al., 2022). Methyltransferase installed the m^6^A modification on mRNA, including methyltransferase-like 3 (METTL3), methyltransferase-like 14 (METTL14), and the Wilms tumor 1-associated protein (WTAP) (Lin et al., 2022; Luo et al., 2022). In addition, the m^6^A modification was wiped by demethylase, including obesity-associated protein (FTO) and alkB homolog 5 (ALKBH5) (Qin et al., 2022; Zhai et al., 2022). In ccRCC, the function of m^6^A has been gradually verified. For example, METTL3 expression is significantly higher compared with adjacent normal tissues in ccRCC tissues and METTL3 depletion significantly inhibits the cell viability, migration and invasion of ccRCC cells (Zhu et al., 2022). Thus, the critical function of m6A modification on ccRCC is obvious.

In present, metabolic alteration is commonly observed in the development of human tumors, including ccRCC. It has been reported that m^6^A modification is significantly involved in tumor processes. In this study, we identified that the novel m6A reader IGF2BP1 up-regulated in the ccRCC and functional analysis determined its oncogenic role in ccRCC. Notably, IGF2BP1 targeted the LDHA mRNA to enhance LDHA mRNA stability, thereby promoting ccRCC aerobic glycolysis. Our findings suggest that IGF2BP1 could be used as a marker to detect cancer energy metabolism and is a potential therapeutic target in ccRCC.

## Materials and Methods

### Cell culture

Human renal proximal tubular epithelial cell line (HK2) and human RCC cell lines (786-O, ACHN) were obtained from the Chinese Academy of Sciences, Shanghai Institutes for Biological Sciences (Shanghai, China). ccRCC cells were maintained in DMEM supplemented with 10% fetal bovine serum (FBS), 2 mM L-glutamine, streptomycin (100 μg/mL), benzylpenicillin (100 U/mL) following standard culture conditions (5% CO_2_) in the humidified environment at 37 °C.

### Vector construction and transfection

To construct stable IGF2BP1 knockdown, the specific targeting to IGF2BP1 was designed with the Invitrogen online tool (https://rnaidesigner.thermofisher.com/rnaiexpress/) and then cloned into the lentiviral pLVX-shRNA vectors (sh-IGF2BP1, sh-NC). The shRNA sequences were as following: sh-IGF2BP1-1, 5′-GCAGTGGTGAATGTCACCTAT-3′; sh-IGF2BP1-2, 5′-CTCCGCTTGTAAGATGATCTT-3′. The stable IGF2BP1 overexpression and negative controls were constructed by GenePharma Biotech Co., LTD (Shanghai, China) (IGF2BP1, vector). The 786-O or ACHN cell lines were cultured in the 6-well plate and then infected with the lentivirus following the manufacturer’s instruction. In 786-O, and ACHN, IGF2BP1 silencing and overexpression were respectively constructed using stable transfection. The 786-O cells received the knockdown and ACHN cells received the overexpression.

### Real‑time qPCR

RNA samples from kidney tumor cells or normal HK-2 cells were extracted using TRIzol kit (cat. 15596026; Invitrogen, Waltham, MA, USA). cDNA was generated using the Reverse Transcription kit (cat. RR047A; Takara, Kusatsu, Japan). Real-time qPCR was performed using SYBR Premix EX Taq kit (cat. RR420A; Takara, Kusatsu, Japan) on ABI7500 (Applied Biosystems, Foster City, CA, USA). The primer pairs’ sequences were listed in Table S1. Gene expression was normalized to actin. Relative quantification levels were determined using the 2^−ΔΔCT^ method.

### Western blot

The total protein in RCC cells was extracted by radioimmunoprecipitation assay (RIPA) lysis buffer containing PMSF. Then, after incubation on ice, the samples were centrifuged at 25,000 g for 10 min with supernatant collected. The protein concentration was measured by bicinchoninic acid (BCA) kit with deionized water. Protein separation was performed *via* 10% SDS-PAGE (Beyotime Biotechnology, Shanghai, China) and then transferred to polyvinylidene fluoride (PVDF) membrane. PVDF membrane was added with Tris-buffered saline tween (TBST) containing 5% skimmed milk for 2 h. Finally, the primary antibody rabbit anti-IGF2BP1 was incubated (antibody IGF2BP1, Cat No. 22803-1-AP; Proteintech, Wuhan, China).

### Glucose uptake, lactate production and ATP analysis

The glycolytic ability of ccRCC cells was calculated using glucose uptake, lactate production and ATP analysis. Firstly, the glucose uptake level was measured using a glucose assay kit (Sigma, St-Louis, MO, USA) as previously described (Wang & Wang, 2022). Then, the lactate production level was detected by a lactate assay kit (Sigma, St-Louis, MO, USA). Lastly, the ATP analysis was performed using the Fluorometric Assay Kit (BioVision, Milpitas, CA, USA).

### Measurement of extracellular acidification rate (ECAR)

ECAR was analyzed using the XF96 Bioenergetic Analyzers (Seahorse Bioscience, Mountain View, CA, USA). All details were prepared and performed according to the manufacturers’ instructions. In brief, sequential injection of glucose (10 mM), oligomycin (1 mM) and 2-deoxyglucose was conducted (50 mM). ECAR measurements was normalized to total protein content and showed as mpH/min.

### RNA immunoprecipitation (RIP) PCR

The RIP experiment was carried using EZ-Magna RIP Kit (Millipore, Billerica, MA, USA) according to manufacturer’s protocol using antibody (5 mg, antibody IGF2BP1, Cat No. 22803-1-AP; Proteintech, Wuhan, China). The 786-O and ACHN cells were lysed in completed RIP lysis buffer. The cellular extraction was incubated with protein A/G agarose beads conjugated with antibodies or control IgG at 4 °C for 2 h. Beads were eluted and incubated with Proteinase. Lastly, purified RNA was subjected to qRT-PCR analysis.

### RNA decay assay for mRNA stability

ccRCC cells were treated with actinomycin D (0.2 mM, cat. GC16866; GlpBio, Montclair, CA, USA) for 30 min and were designated as the 0 h samples. The 0, 3 and 6 h samples were collected for RNA extraction. Then, cDNA was synthesized *via* reverse transcription and then performed using oligo(dT) primer. The quantitated RNA level was determined by RT-qPCR. Half-life (t_1/2_) of mature LDHA mRNA was calculated using ln_2_/slope and actin as normalization.

### RNA m^6^A methylation quantification

As previously reported, the m^6^A RNA Methylation Assay Kit (cat. ab185912; Abcam, Cambridge, UK) was performed to evaluate the content of m^6^A in total RNA. In brief, RNA (300 ng) was coated in assay wells accompanied with m^6^A standard protocols. In solution, capture antibody and secondary detection antibody solution were added. Then, the colorimetric m^6^A levels were quantified by assessing absorbance at OD450 based on the standard curve.

### Statistical and data analysis

All data in present study were processed using GraphPad Prism 9.0 and expressed as mean ± standard deviation (SD). Two-sided unpaired Student’s t-test and one-way analysis of variance Tukey’s *post-hoc* test were utilized for two-group comparisons or multiple group comparisons. *p*-value < 0.05 was considered statistically significant. All *in vitro* experiments were performed in triplicate and were repeated three times.

## Results

### IGF2BP1 up-regulated in the ccRCC

**Figure 1:**
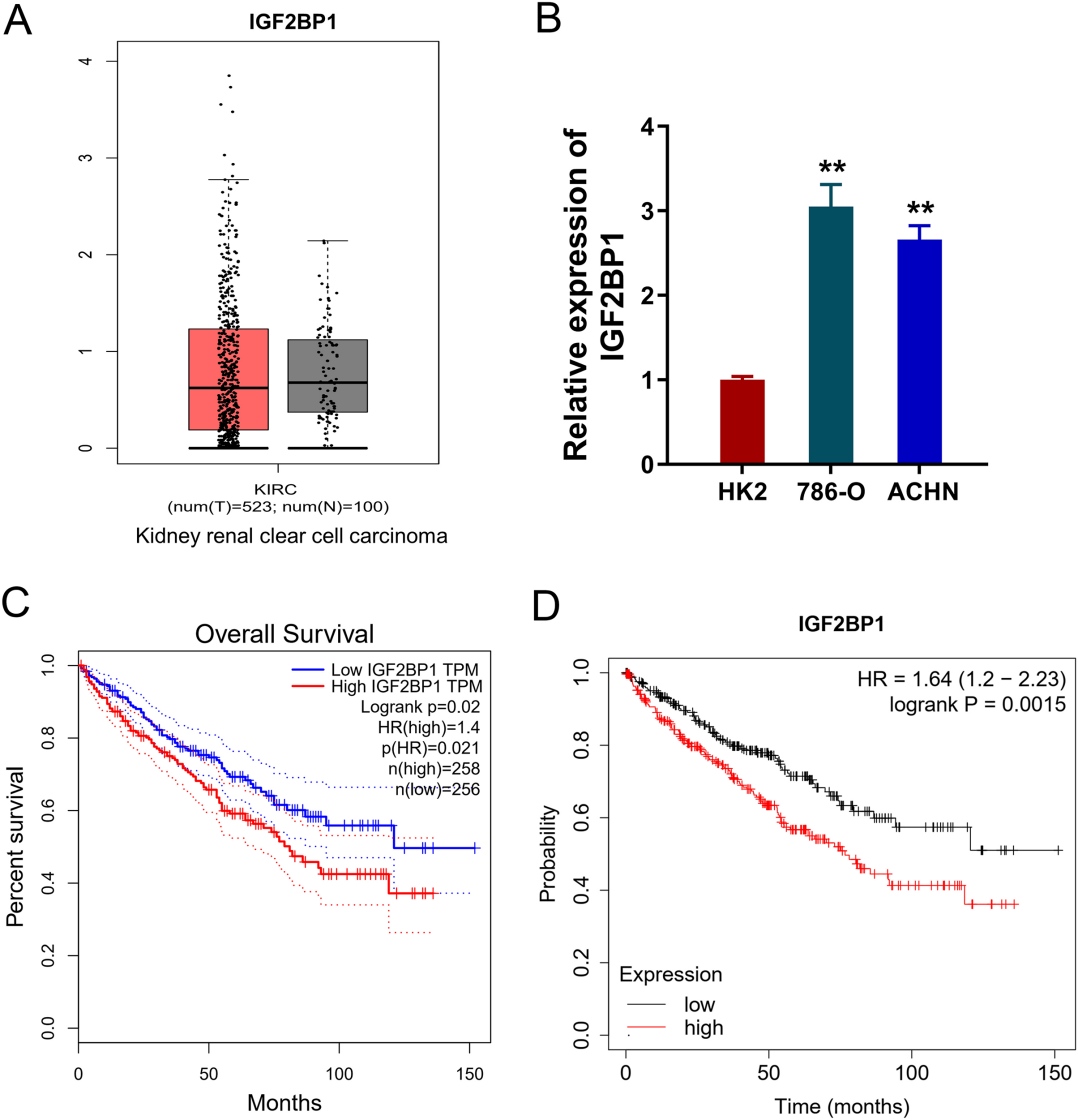
IGF2BP1 up-regulated in the ccRCC. (A) Landscape analysis by ATGC-GEPIA (http://gepia.cancer-pku.cn/index.html) revealed the enrichment level of IGF2BP1 in the kidney renal clear cell carcinoma (KIRC) tissue and normal tissue by Match TCGA normal and GTEx data. (B) IGF2BP1 mRNA level was detected by RT-PCR analysis in human renal proximal tubular epithelial cell line (HK2) and human RCC cell lines (786-O, ACHN). The RT-PCR experiments were performed in triplicate. (C) ATGC-GEPIA (http://gepia.cancer-pku.cn/index.html) revealed the prognosis of kidney renal clear cell carcinoma (KIRC) patients with higher/lower IGF2BP1 level. (D) Kaplan-Meier Plotter analysis revealed the worse survival prognosis of KIRC patients with a higher IGF2BP1 level. **p < 0.01.

In the ccRCC tissue cohort, landscape analysis by ATGC (http://gepia.cancer-pku.cn/index.html) revealed the level of IGF2BP1 in the kidney renal clear cell carcinoma (KIRC) tissue compared to normal tissue by ‘Match TCGA normal and GTEx data (Fig. 1A). In ccRCC cells, RT-PCR analysis revealed that IGF2BP1 mRNA level increased compared to normal cells (*p* < 0.05, Fig. 1B). Besides, the same landscape analysis also revealed that the prognosis of ccRCC patients with higher IGF2BP1 level was significantly lower than that of lower IGF2BP1 level (Fig. 1C). Moreover, Kaplan-Meier Plotter analysis found that the higher IGF2BP1 level indicated worse survival prognosis (Fig. 1D). Therefore, these data illustrated that IGF2BP1 up-regulated in the ccRCC.

### IGF2BP1 promoted the aerobic glycolysis of ccRCC

**Figure 2:**
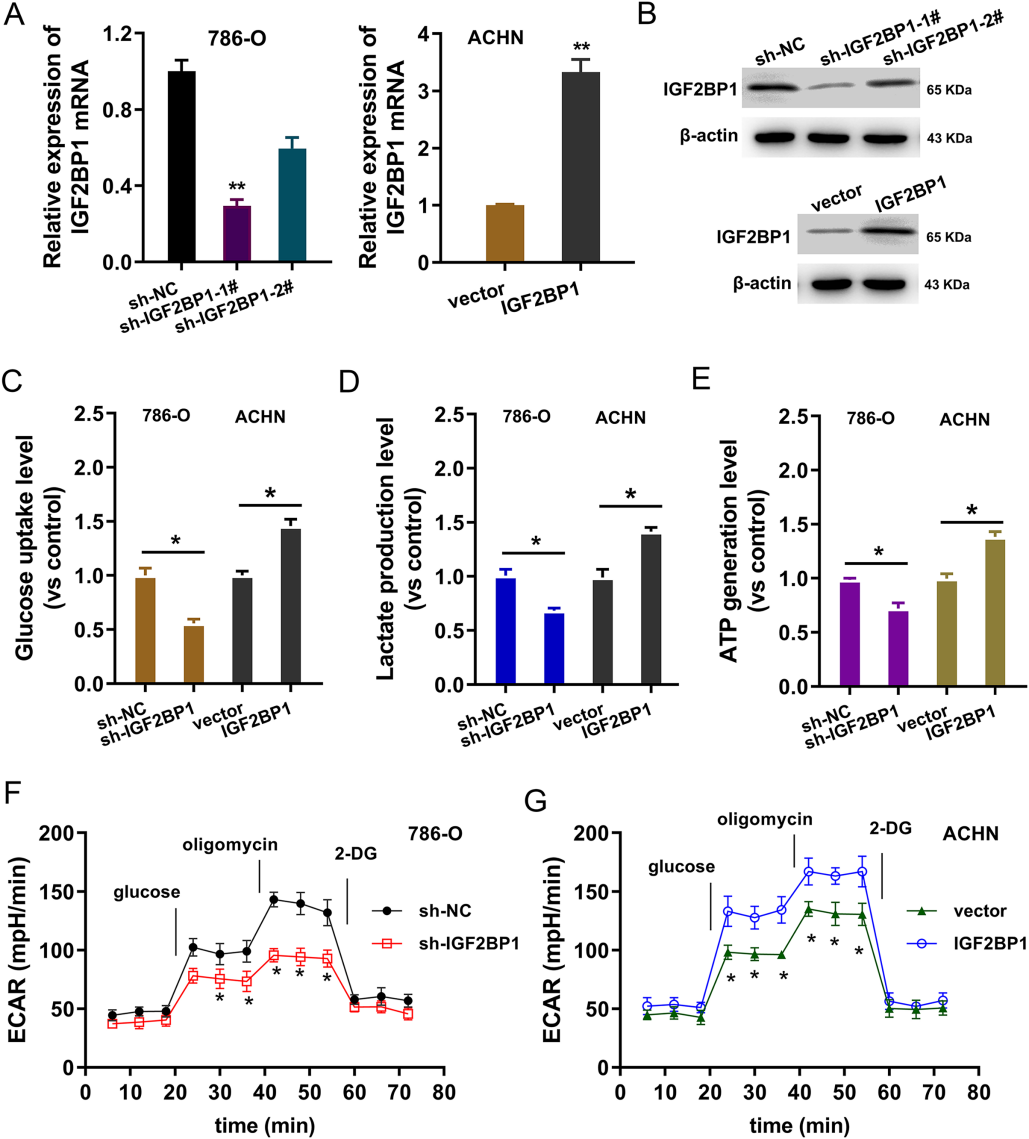
IGF2BP1 promoted the aerobic glycolysis of ccRCC. (A) RT-PCR and (B) western blot analysis was performed in the ccRCC cells (786-O, ACHN) with stable transfection of IGF2BP1 silencing and overexpression. (C) Glucose uptake analysis was performed using ccRCC cells (786-O, ACHN) with IGF2BP1 overexpression/silencing to detect the glucose uptake. shRNA-1# was used for the silencing. (D) Lactate production was performed using ccRCC cells (786-O, ACHN) with IGF2BP1 overexpression/silencing to detect the lactate production quantity. (E) ATP generation analysis was performed using ccRCC cells (786-O, ACHN) with IGF2BP1 overexpression/silencing to detect the ATP level. (F and G) The glycolysis rate and glycolytic capacity in 786-O or ACHN cells were detected using extracellular acidification rate (ECAR) analysis. **p < 0.01; *p < 0.05. All these experiments were performed in triplicate and were repeated three times.

In the ccRCC cells (786-O, ACHN), IGF2BP1 silencing and overexpression were respectively constructed and the transfection efficient was detected using RT-PCR and western blot (*p* < 0.025, Figs. 2A and 2B). To detect the bio-functions of IGF2BP1, cellular assays were performed and results showed that IGF2BP1 could remarkably regulate the energy metabolism (aerobic glycolysis). Firstly, we detected the glucose uptake, lactate production and ATP generation in ccRCC cells. Results indicated that silencing of IGF2BP1 (shRNA-1#) repressed the glucose uptake (*p* < 0.032, Fig. 2C), lactate production (*p* < 0.013, Fig. 2D) and ATP generation (*p* < 0.016, Fig. 2E) in 786-O cells.. Besides, the overexpression of IGF2BP1 promoted the glucose uptake (*p* < 0.024, Fig. 2C), lactate production (*p* < 0.011, Fig. 2D) and ATP generation (*p* < 0.009, Fig. 2E) in ACHN cell. Furthermore, the extracellular acidification rate (ECAR) analysis was detected and results demonstrated that silencing of IGF2BP1 repressed the glycolysis rate in 786-O cell (*p* < 0.044, Fig. 2F), while overexpression of IGF2BP1 promoted the glycolytic capacity (*p* < 0.035, Fig. 2G). Therefore, these data illustrated that IGF2BP1 promoted the aerobic glycolysis of ccRCC.

### LDHA acted as the target modified by IGF2BP1 *via* m^6^A binding 

**Figure 3:**
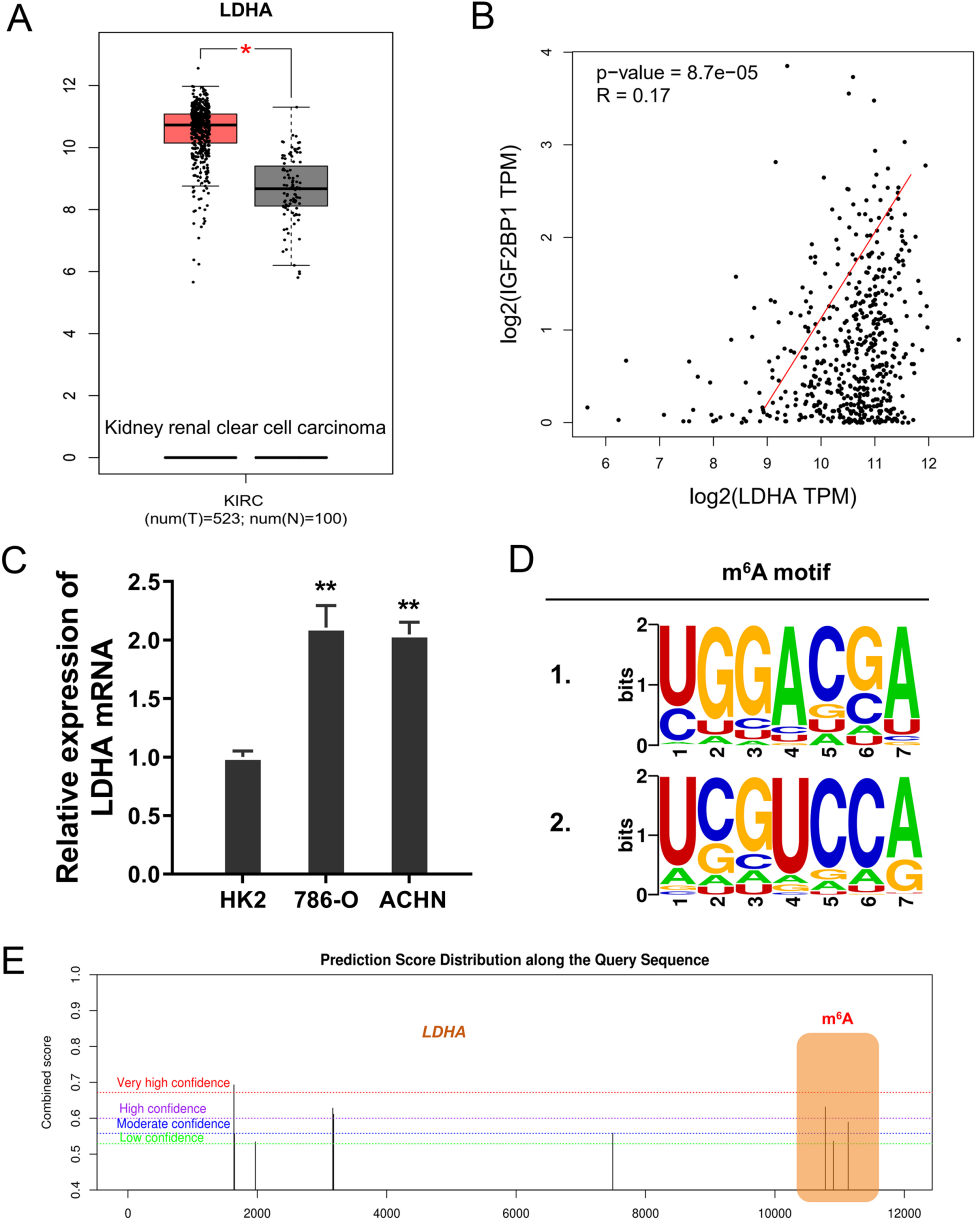
LDHA acted as the target modified by IGF2BP1 *via* m^6^A binding. (A) Landscape analysis by ATGC (http://gepia.cancer-pku.cn/index.html) revealed the LDHA level in the kidney renal clear cell carcinoma (KIRC) tissue compared to normal tissue. (B) Interaction analysis revealed the positive correlation within LDHA and IGF2BP1 level in KIRC patients. (C) The level of LDHA mRNA was detected by RT-PCR in ccRCC cells (786-O, ACHN). (D) Online predictive analysis (https://rna.sysu.edu.cn/rmbase/) revealed the m^6^A motif as ‘GGACA’. (E) The genomic analysis using SRAMP (http://www.cuilab.cn/sramp) showed the m^6^A modification site main location in the 3′-UTR of LDHA mRNA. **p < 0.01; *p < 0.05.

In the ccRCC tissue cohort, landscape analysis by ATGC (http://gepia.cancer-pku.cn/index.html) revealed that LDHA up-regulated in the kidney renal clear cell carcinoma (KIRC) tissue compared to normal tissue (Fig. 3A). Interaction analysis revealed that LDHA was positively correlated to the level of IGF2BP1 in KIRC patients (Fig. 3B). In ccRCC cells (786-O, ACHN), the level of LDHA also significantly up-regulated (Fig. 3C). Online predictive analysis revealed that the m^6^A motif was predicted and analyzed as ‘GGACA’ (Fig. 3D). Moreover, the genomic analysis using SRAMP (http://www.cuilab.cn/sramp) showed that the m^6^A modification site mainly located in the 3′-UTR of LDHA mRNA (Fig. 3E). Therefore, these data illustrated that LDHA acted as the target modified by IGF2BP1 *via* m^6^A binding.

### IGF2BP1 enhanced the stability of LDHA mRNA

**Figure 4:**
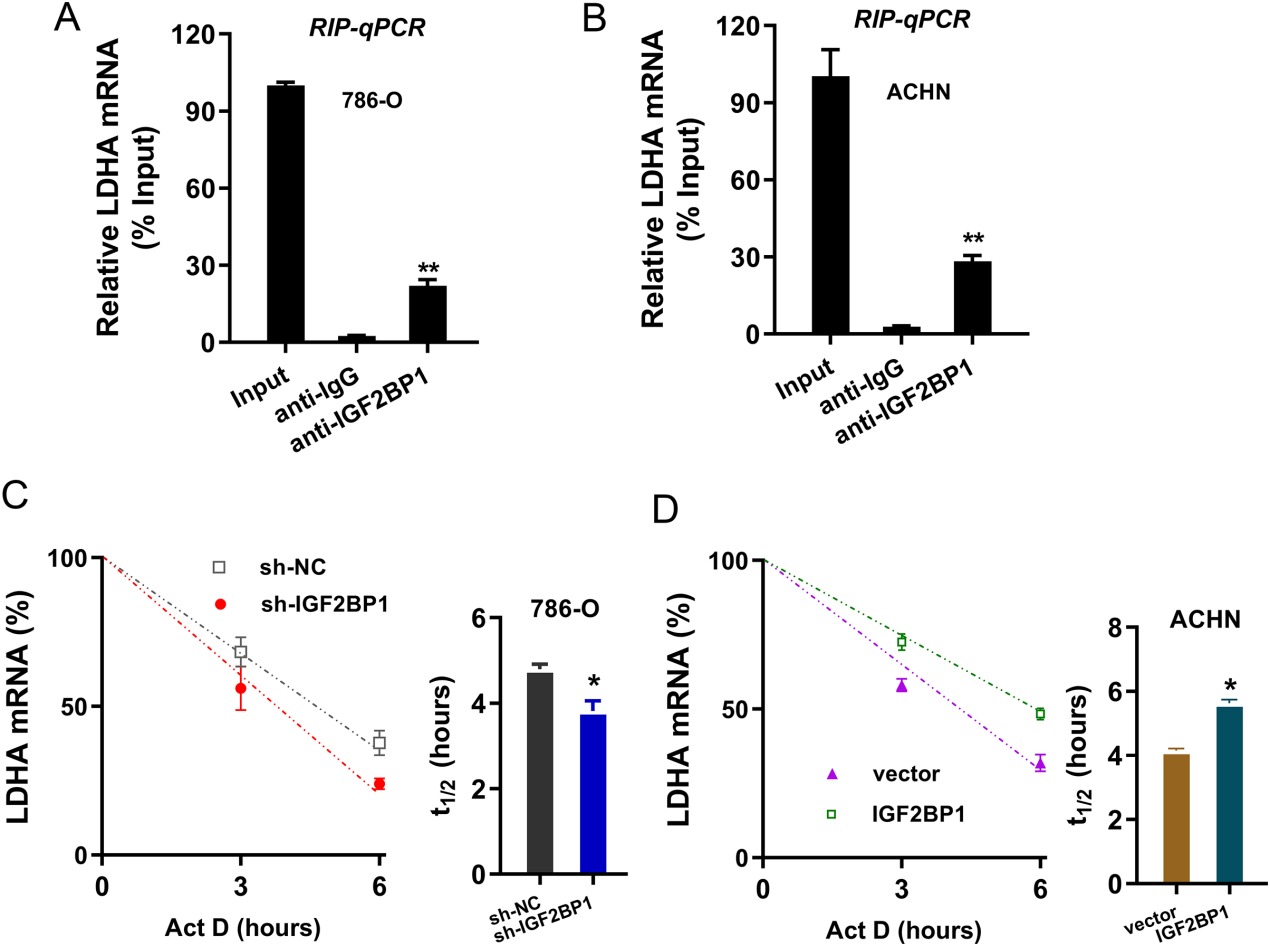
IGF2BP1 enhanced the stability of LDHA mRNA. (A and B) The RIP-PCR analysis was performed using anti-IGF2BP1 or anti-IgG to detect the interaction within LDHA and IGF2BP1 in ccRCC cells (786-O, ACHN). (C and D) RNA decay assay was performed using Act D to show the LDHA mRNA remaining level in ccRCC cells (786-O, ACHN) with IGF2BP1 silencing/overexpression. The half-life (t_1/2_) of LDHA mRNA was calculated using ln2/slope and actin was used for normalization. **p < 0.01; *p < 0.05. All these experiments were performed in triplicate and were repeated three times.

Given that we found that LDHA acted as the target of IGF2BP1 in ccRCC, next, we tried to investigate the molecular interaction within LDHA and IGF2BP1. Firstly, the RIP-PCR analysis was performed to detect the interaction within LDHA and IGF2BP1, and results illustrated that LDHA significantly combined with IGF2BP1 in 786-O cells (Fig. 4A) and ACHN cells (Fig. 4B). Because there was positive interaction within LDHA and IGF2BP1, we imagined that IGF2BP1 could positively regulate the LDHA expression. Then, we performed the RNA decay assay to show whether IGF2BP1 could positively regulate the stability of LDHA mRNA. Results illustrated that IGF2BP1 silencing repressed the LDHA mRNA remaining level in 786-O cells (Fig. 4C), while IGF2BP1 overexpression promoted the LDHA mRNA remaining level in ACHN cells (Fig. 4D). Therefore, these data illustrated that IGF2BP1 enhanced the stability of LDHA mRNA.

## Discussion

To date, the progression of RCC is associated with tumorigenesis and metastasis (Chen et al., 2021; Liu et al., 2022a). m^6^A acts as the most abundant modification in eukaryocyte mRNA, which is regulated by m^6^A methyltransferases, demethylases and readers (Guimarães-Teixeira et al., 2021; He et al., 2021). Here, we tried to investigate the potential roles of novel m^6^A reader IGF2BP1 in ccRCC.

Emerging evidences have suggested that the m^6^A modifications are associated with human cancer proliferation, invasion and metastasis (Akhtar, Lugoboni & Junion, 2021; Li et al., 2021). From this research point, we could explore the deep mechanism of ccRCC. Results indicated that the expression of m^6^A reader IGF2BP1 elevated in ccRCC and functionally necessary for the aerobic glycolysis by regulating glucose uptake, lactate production and ATP level. Functional assays using loss/gain-functional assays revealed the functions of IGF2BP1 on ccRCC energy metabolism.

Currently, targeted therapies are the standard treatment options for renal cell carcinoma, functioning as an oncogene or anti-oncogene in malignant tumors (Relier, Rivals & David, 2022; Yang et al., 2022). On this basis of epigenetics modification, this regulatory effects of IGF2BP1 suggested that m^6^A could be utilized to develop new strategies for targeted therapies. However, among several well-known key enzymes which potentially promote mRNA stability, several m^6^A regulators was predicatively identified in renal cancers. For example, m^6^A writers METTL14 expression significantly down-regulates in ccRCC tissues, and METTL14 upregulation inhibits ccRCC’s proliferation and migration *via* activating the PI3K/AKT signalling pathway. METTL14 enhances the Pten mRNA stability through YTHDF1/m^6^A/dependent (Zhang, Luo & Qiao, 2022). Besides, depletion of METTL3 significantly inhibits the cell viability, migration and invasion abilities of ccRCC cell lines through regulating HHLA2 expression *via* m^6^A modification of HHLA2 mRNA (Zhu et al., 2022). Thus, these findings reveal that m^6^A modification could regulate the renal cancer progression.

Here, we found that m^6^A reader IGF2BP1 elevated in ccRCC and promoted the aerobic glycolysis, including glucose uptake, lactate production and ATP level. The aerobic glycolysis is critical for the ccRCC tumorigenesis. Particularly, cancer cells tend to have elevated levels of glycolysis, reducing glucose to lactate. Glycolysis may be anaerobic or aerobic, aerobic glycolysis or the Warburg effect. LDHA is a critical regulator in tumor aerobic glycolysis, and critical literature reveals its critical function in tumorigenesis. For example, LDHA is overexpressed in RCC tissues and predicts worse survival, and LDHA downregulation suppresses RCC cells migration and invasion and the Warburg effect (Zhao et al., 2017).

Mechanistically, we found that enforced IGF2BP1 expression in ccRCC enhanced LDHA mRNA expression directly, as well as that of aerobic glycolysis. IGF2BP1 could exert its regulatory roles *via* binding targets through transcript’s stability. For example, in gastric cancer, m^6^A reader IGF2BP1 upregulated in gastric cancer tissue and acted as a predictor of poor prognosis for gastric cancer patients and IGF2BP1 directly interacts with c-MYC mRNA *via* m^6^A-dependent manner to stabilize its stability (Luo & Lin, 2022). In lipopolysaccharide-induced cardiomyocytes, m^6^A reader IGF2BP1 recognizes the m^6^A modified sites on HDAC4 mRNA and enhances its mRNA stability (Shen et al., 2022). Thus, the critical role of IGF2BP1 might promote this tumor progression.

**Figure 5:**
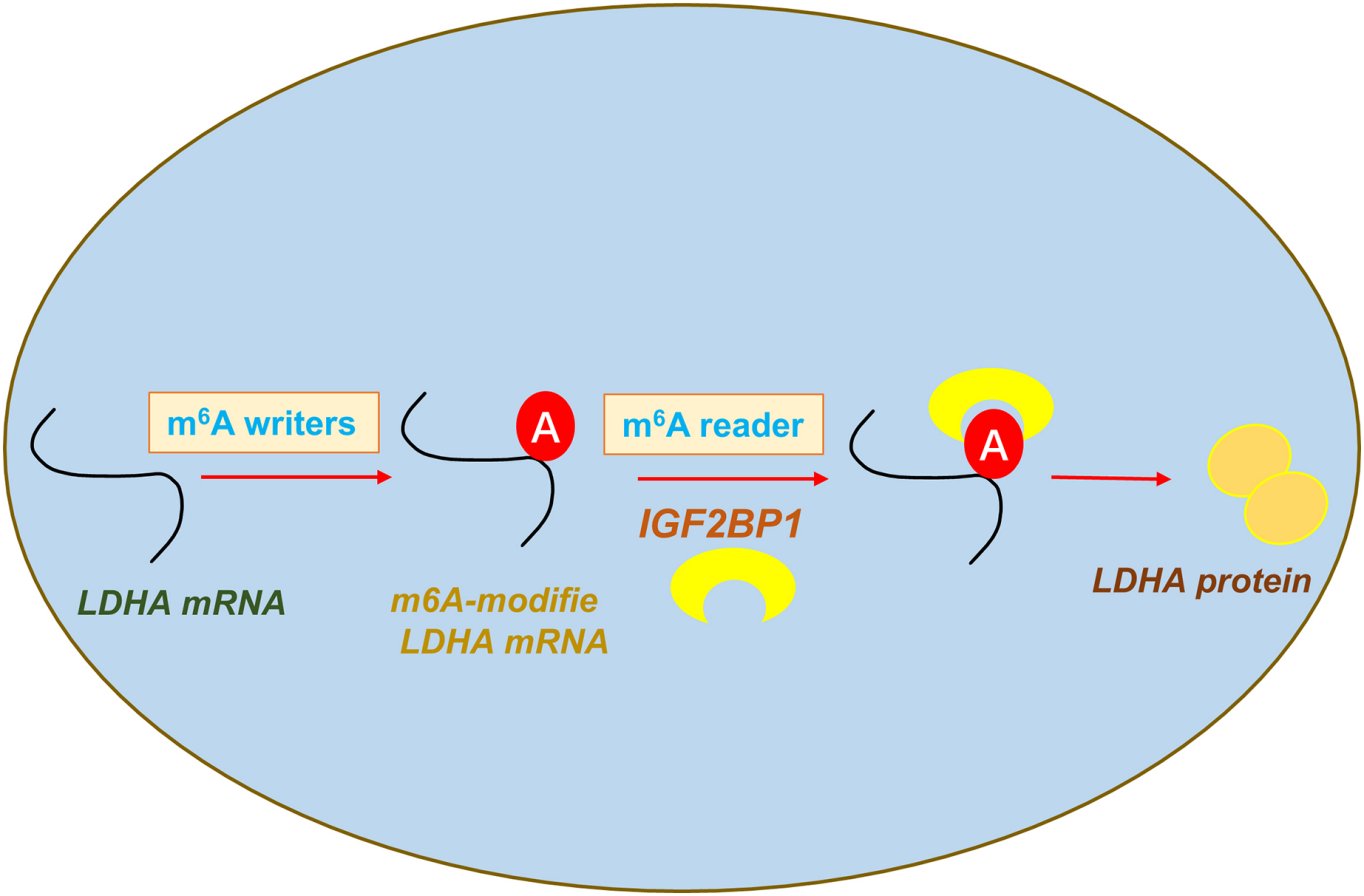
IGF2BP1 targets LDHA to promote the aerobic glycolysis of ccRCC *via* m^6^A-dependent manner.

In conclusion, our data shows that an oncogenic m^6^A reader IGF2BP1 promotes ccRCC aerobic glycolysis and malignant phenotype. Moreover, we identified that IGF2BP1 can directly bind LDHA and induce its mRNA stability increasing. In addition, we reported a regulatory mechanism that IGF2BP1 targets LDHA to promote the aerobic glycolysis of ccRCC *via* m^6^A-dependent manner (Fig. 5).

## Supplemental Information

10.7717/peerj.14591/supp-1 Supplemental Information 1 PCR primers.

10.7717/peerj.14591/supp-2 Supplemental Information 2 Raw data.

10.7717/peerj.14591/supp-3 Supplemental Information 3 Uncropped blot.

## Additional Information and Declarations

### Data Availability

The following information was supplied regarding data availability: The raw data is available in the Supplemental File.
