# Supplementary material for: N6-methyladenosine (m6A) reader IGF2BP1 facilitates clear-cell renal cell carcinoma aerobic glycolysis
Source: PeerJ. 2023 Jan 18;11:e14591. doi: 10.7717/peerj.14591 (PMC9864111; doi:10.7717/peerj.14591)
Supplement: Supplemental Information 1 [file peerj-11-14591-s001.docx]

**Table S1**. Primers sequences for qRT-PCR and sequences of shRNA.

|  | Sequences |
| --- | --- |
| IGF2BP1 | forward, 5’-GGCCATCGAGAATTGTTGCAG-3’  reverse, 5’-CCAGGGATCAGGTGAGACTG-3’ |
| LDHA | forward, 5’-TTGACCTACGTGGCTTGGAAG-3’  reverse, 5’-GGTAACGGAATCGGGCTGAAT-3’ |
| sh-IGF2BP1-1 | 5’- GCAGTGGTGAATGTCACCTAT-3’ |
| sh-IGF2BP1-2 | 5’-CTCCGCTTGTAAGATGATCTT-3’ |
| Actin | forward, 5’-CATGTACGTTGCTATCCAGGC-3’  reverse, 5’-CTCCTTAATGTCACGCACGAT-3’ |
