# Supplementary figures and images for: N6-methyladenosine (m6A) reader IGF2BP1 facilitates clear-cell renal cell carcinoma aerobic glycolysis
Source: PeerJ. 2023 Jan 18;11:e14591. doi: 10.7717/peerj.14591 (PMC9864111; doi:10.7717/peerj.14591)

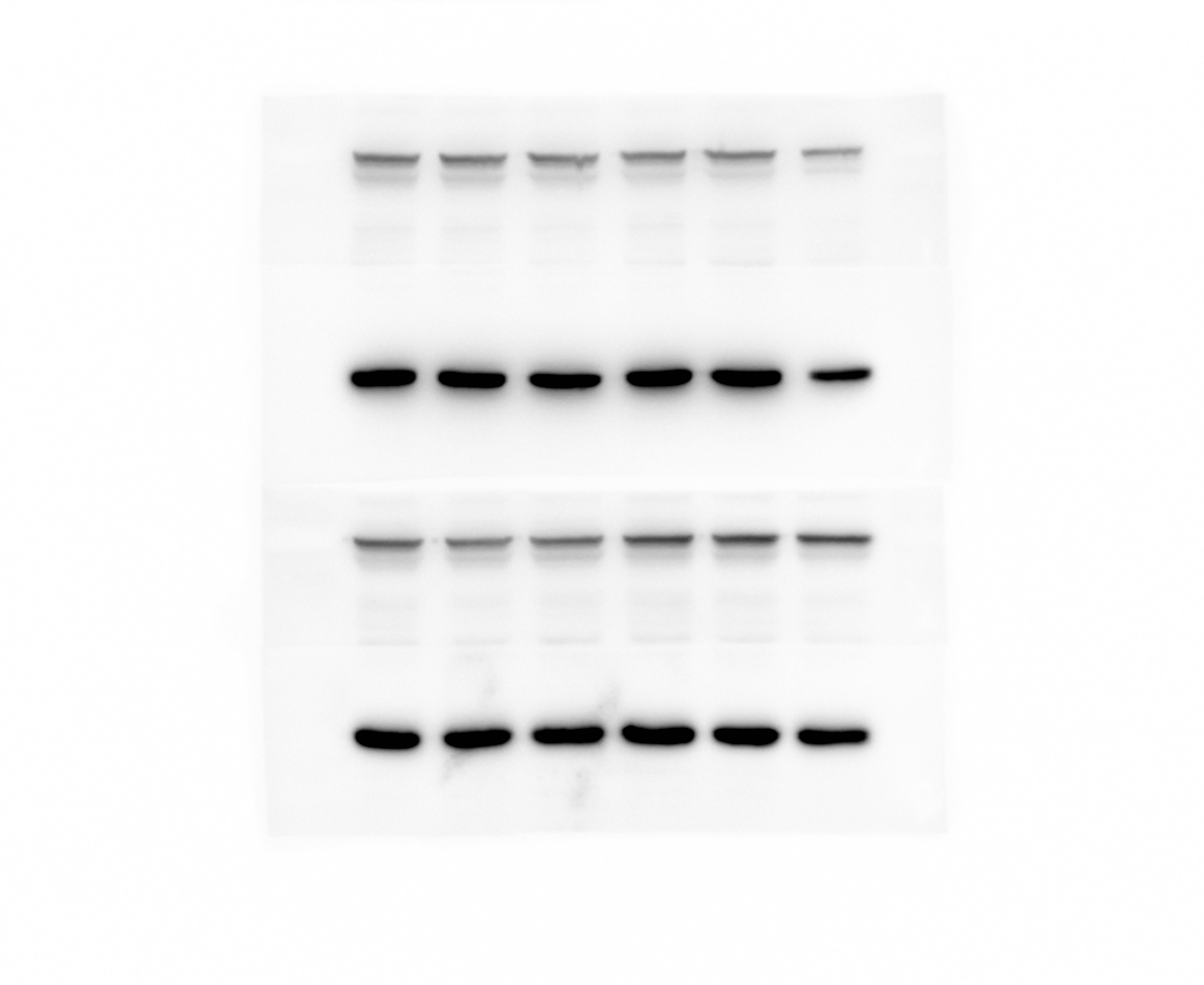

Supplement: Supplemental Information 3 [file peerj-11-14591-s003.zip › uncropped blot/Fig 2-1 .TIF]

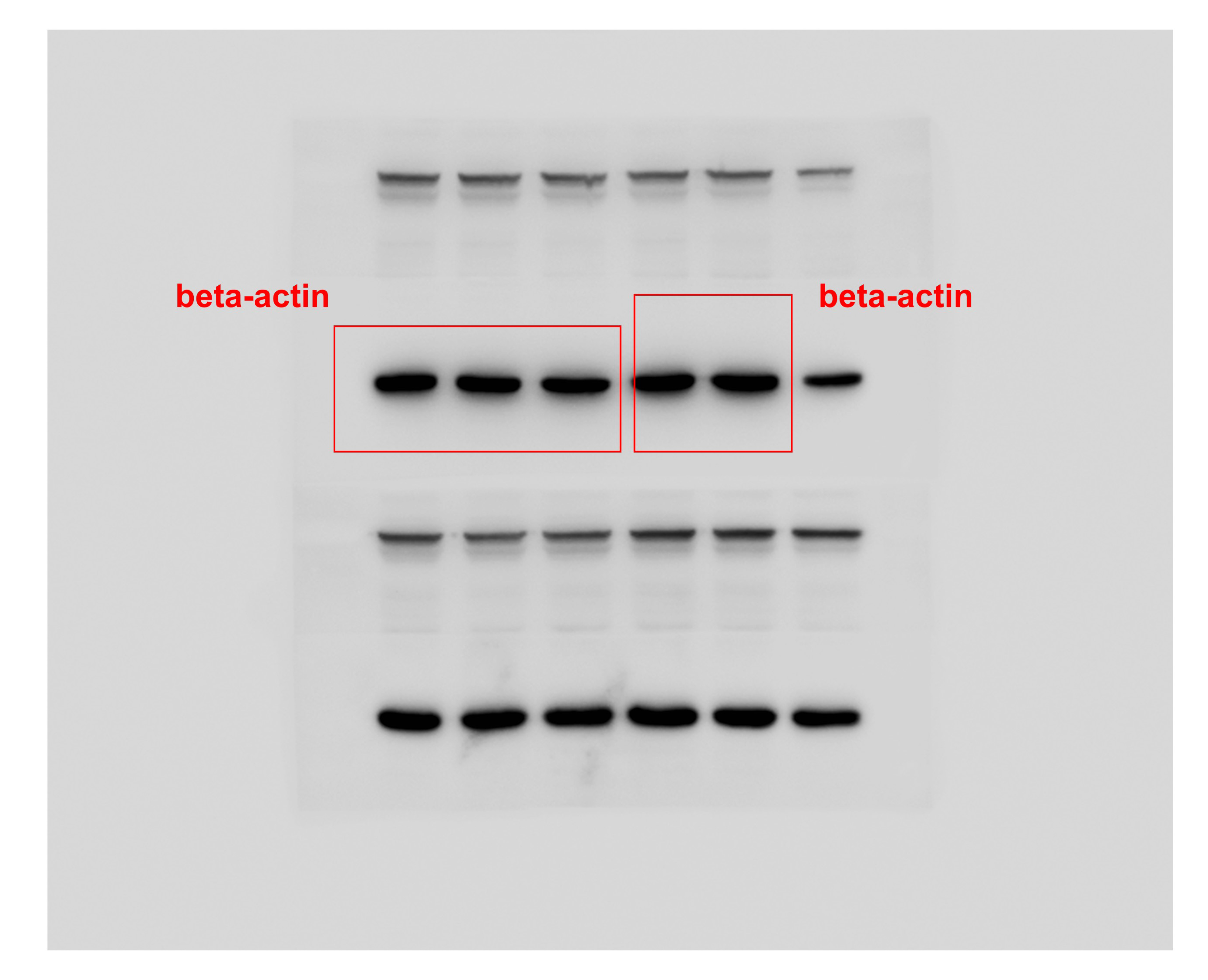

Supplement: Supplemental Information 3 [file peerj-11-14591-s003.zip › uncropped blot/Fig 2-1 .jpg]

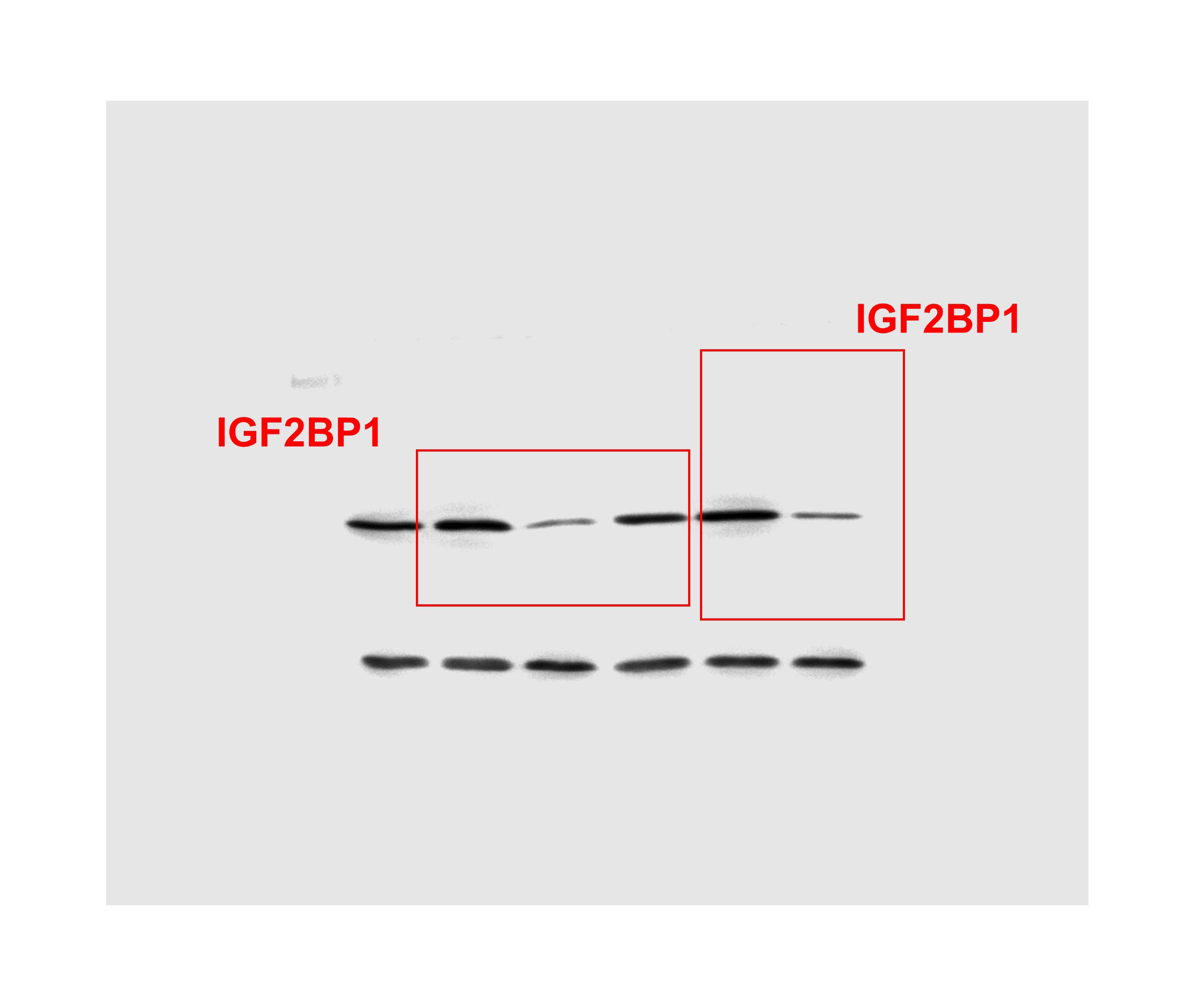

Supplement: Supplemental Information 3 [file peerj-11-14591-s003.zip › uncropped blot/Fig 2-2 .jpg]

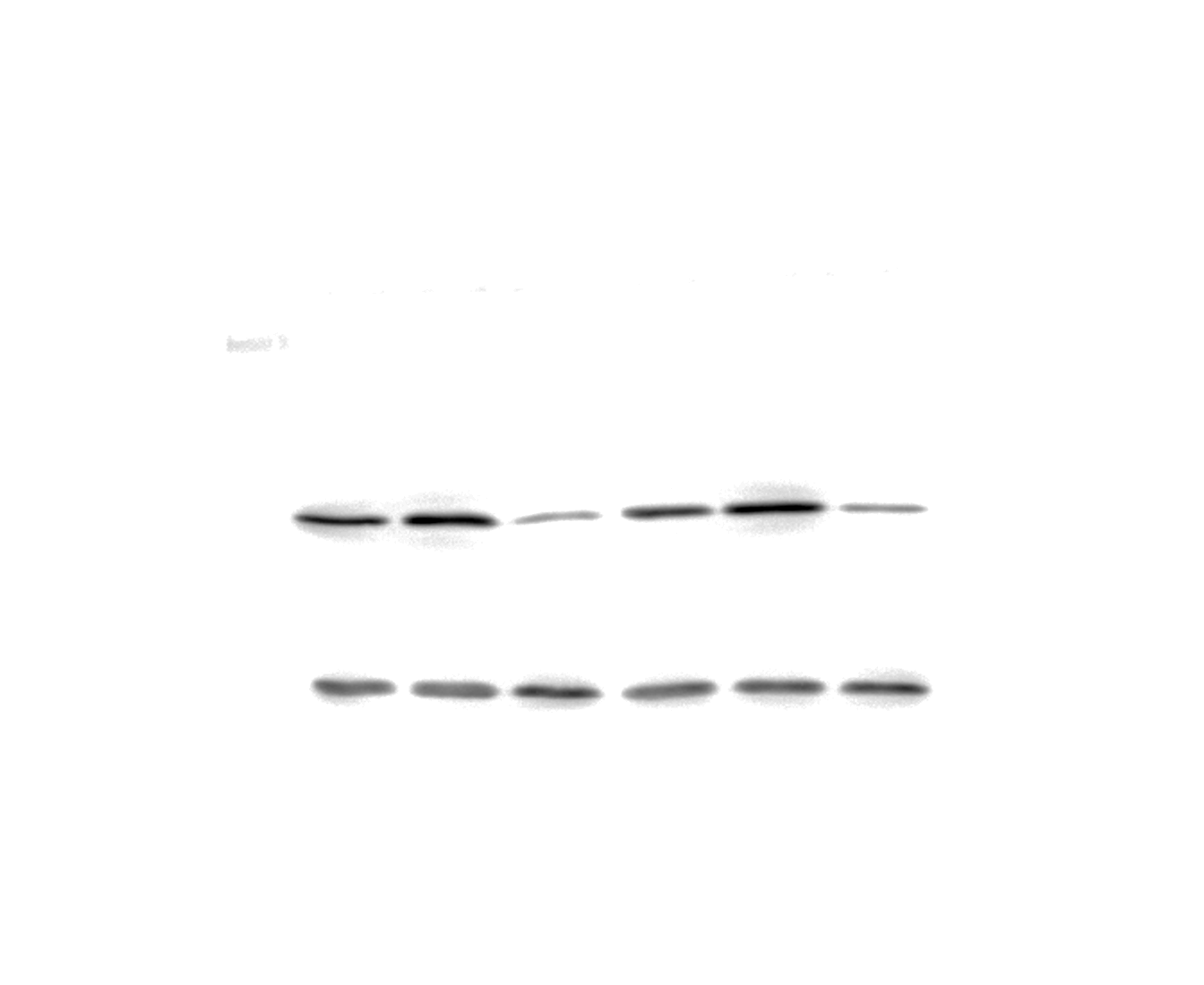

Supplement: Supplemental Information 3 [file peerj-11-14591-s003.zip › uncropped blot/Fig 2-2.Tif]

bits

2

1

0

1

2

3

4

5

6

7

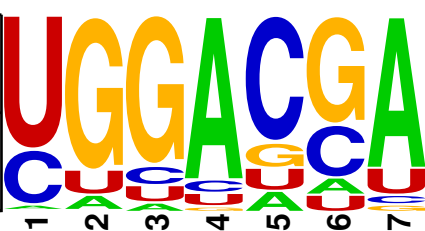

Supplement: Supplemental Information 3 [file peerj-11-14591-s003.zip › uncropped blot/Human_m6A_GSM2203056_motif1.logoxy.pdf]

bits

2

1

0

1

2

3

4

5

6

7

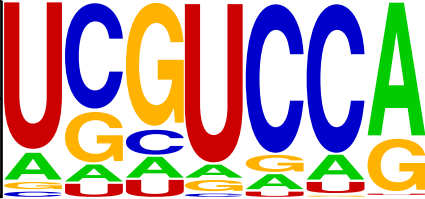

Supplement: Supplemental Information 3 [file peerj-11-14591-s003.zip › uncropped blot/Human_m6A_GSM2203056_motif1.rvlogoxy.pdf]

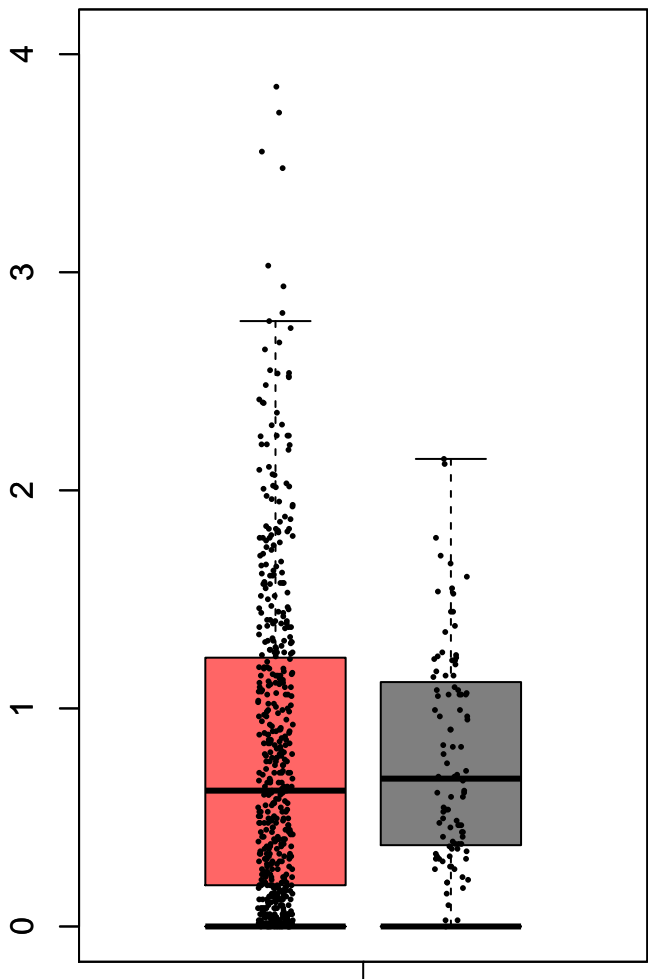

KIRC  
(num(T)=523; num(N)=100)

Supplement: Supplemental Information 3 [file peerj-11-14591-s003.zip › uncropped blot/IGF2BP1_boxplot_M0hEN.pdf]

# Overall Survival

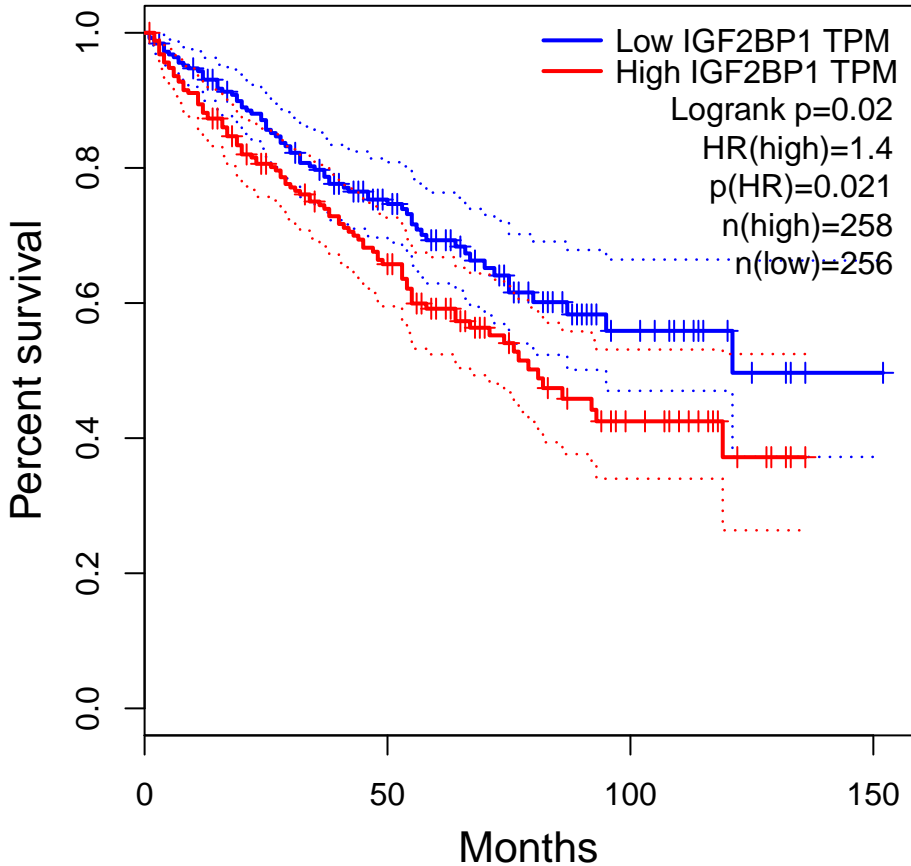

Supplement: Supplemental Information 3 [file peerj-11-14591-s003.zip › uncropped blot/IGF2BP1_survival_gM9qP.pdf]

p-value =  $8.7\text{e-}05$   
R = 0.17

log2(IGF2BP1 TPM)

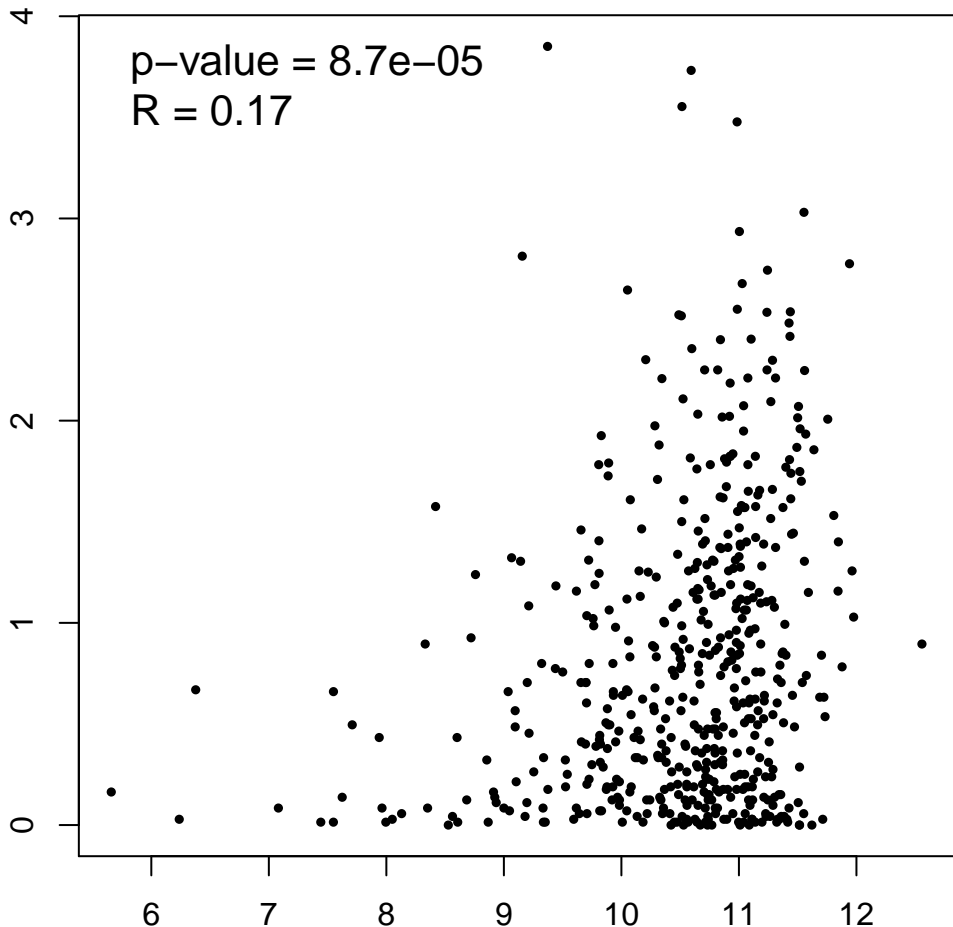

log2(LDHA TPM)

Supplement: Supplemental Information 3 [file peerj-11-14591-s003.zip › uncropped blot/LDHA_IGF2BP1_correlation_EYKIy.pdf]

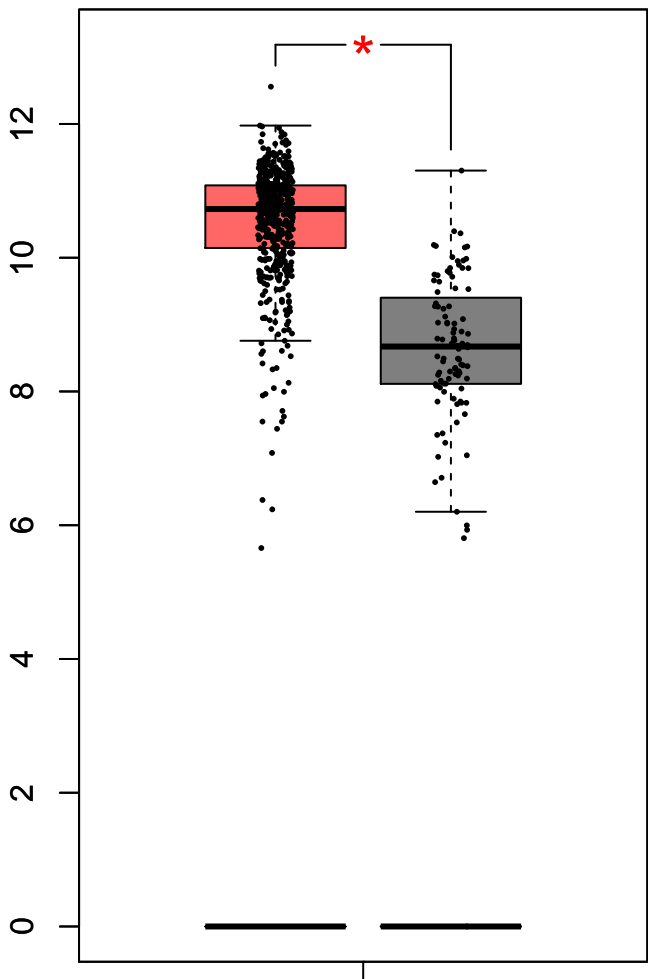

KIRC  
(num(T)=523; num(N)=100)

Supplement: Supplemental Information 3 [file peerj-11-14591-s003.zip › uncropped blot/LDHA_boxplot_z2Mak.pdf]

# IGF2BP1

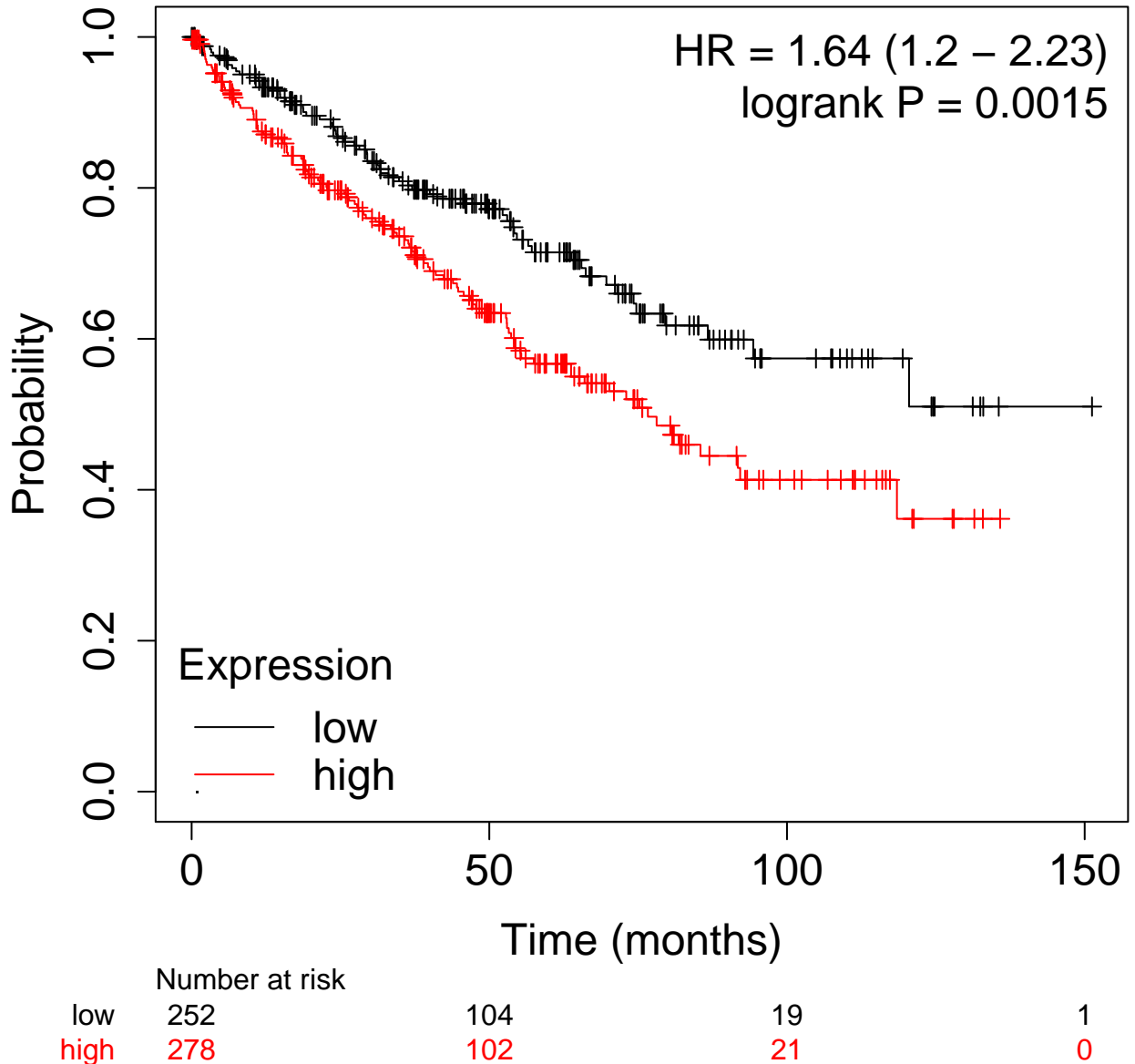

Supplement: Supplemental Information 3 [file peerj-11-14591-s003.zip › uncropped blot/km_220729_124241_853100_62e3d5c1d049d_IGF2BP1.pdf]
